# Supplementary material for: Sustained-input switches for transcription factors and microRNAs are central building blocks of eukaryotic gene circuits
Source: Genome Biol. 2013 Aug 23;14(8):R85. doi: 10.1186/gb-2013-14-8-r85 (PMC4054853; doi:10.1186/gb-2013-14-8-r85)
Supplement: Additional file 5 — HTML Browsable Motif Output. Zipped folder containing all WaRSwap and FANMOD motif output, viewable in a web browser. [file gb-2013-14-8-r85-S5.ZIP › HTML_browsable_motif_output/WaRSwap_ath_tair10_withSelfLoops/sigs_WSWRSLDraw_TAIR10_SLColors-2500.pvals.heatmaps.html/motif_id_38_011020013_tftype_ath_upstream_-3000_0.html]

```
BG_MODEL = WARSwap
MOTIF_ID = 38_011020013
TF_TYPE = ath
UPSTREAM = -3000_0


PVals
FNR = 0.2	FNR = 0.4	FNR = 0.6	FNR = 0.8
deltaG = 60	0.773	0.915	0.001	0
deltaG = 70	0.777	0.93	0	0
deltaG = 80	0.769	0.925	0.002	0

ZScores
FNR = 0.2	FNR = 0.4	FNR = 0.6	FNR = 0.8
deltaG = 60	-0.952	-1.412	3.181	3.267
deltaG = 70	-0.946	-1.462	3.123	3.392
deltaG = 80	-0.943	-1.44	3.084	3.306

StDevs
FNR = 0.2	FNR = 0.4	FNR = 0.6	FNR = 0.8
deltaG = 60	138536.128	60068.755	13866.689	3337.784
deltaG = 70	137683.677	58676.197	13979.578	3231.504
deltaG = 80	138232.826	59253.408	13968.01	3314.777
```
